# Steady-state equation of water vapor sorption for CaCl_2_-based chemical sorbents and its application

**DOI:** 10.1038/srep34115

**Published:** 2016-09-29

**Authors:** Haiquan Zhang, Yanping Yuan, Qingrong Sun, Xiaoling Cao, Liangliang Sun

**Affiliations:** 1 School of Mechanical Engineering, Southwest Jiaotong University, 610031 Chengdu, China

## Abstract

Green CaCl_2_-based chemical sorbent has been widely used in sorption refrigeration, air purification and air desiccation. Methods to improve the sorption rate have been extensively investigated, but the corresponding theoretical formulations have not been reported. In this paper, a sorption system of solid-liquid coexistence is established based on the hypothesis of steady-state sorption. The combination of theoretical analysis and experimental results indicates that the system can be described by steady-state sorption process. The steady-state sorption equation, *μ* = (*η* − γ_T_) 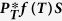, was obtained in consideration of humidity, temperature and the surface area. Based on engineering applications and this equation, two methods including an increase of specific surface area and adjustment of the critical relative humidity (γ) for chemical sorbents, have been proposed to increase the sorption rate. The results indicate that the CaCl_2_/CNTs composite with a large specific surface area can be obtained by coating CaCl_2_ powder on the surface of carbon nanotubes (CNTs). The composite reached sorption equilibrium within only 4 h, and the sorption capacity was improved by 75% compared with pure CaCl_2_ powder. Furthermore, the addition of NaCl powder to saturated CaCl_2_ solution could significantly lower the solution’s *γ*. The sorption rate was improved by 30% under the same environment.

Sustainable water supply is important for social and economic development. However, fresh water that can be directly used has been exhausting rapidly due to gradual natural environmental degradation, especially the serious pollution of lakes and rivers^1^,^2^,^3^,^4^. The atmosphere stores approximately 14, 000 km^3^ fresh water, 10 times more than the surface fresh water on the Earth^5^. Obtaining sustainable fresh water from air has attracted much attention of many researchers. At present, methods of mechanical compression/condensation^6^,^7^ and adsorption^8^,^9^,^10^,^11^ are frequently-used to obtain fresh water from air. The adsorption of water vapour from atmosphere has been widely researched due to its high efficiency, low energy consumption, friendliness to the environment, low-cost and renewable characteristics. The common sorbents are physical^12^,^13^,^14^,^15^ and chemical sorbents^16^,^17^. Chemical adsorbents present large adsorption capacities and thermal energy.

Among the various chemical sorbents, green CaCl_2_ has been widely applied to air desiccation, thermal energy storage, and gas purification, because of its large water adsorption capacity, excellent chemical and thermal stability, excellent cycle performance, low cost, and absence of environmental pollution. For the other chemical sorbents, LiCl with crystal water produces HCl when drying temperature is over 98 °C; when the environment temperature rises up to 132 °C, Ca(NO_3_)_2_ decomposes quickly, producing oxygen and carcinogenic nitrite; CuSO_4_ and MgSO_4_ display a low water uptake. It means that the reusability of the CaCl_2_-based chemical sorbents can effectively reduce the overall cost of the sorbent for practical application in comparison with other chemical sorbents.

In the adsorption process, pure CaCl_2_ can generate CaCl_2_·0.33H_2_O, CaCl_2_·H_2_O, CaCl_2_·2H_2_O, and many other types of stable hydrates. Its equilibrium water adsorption capacity exceeds 100 g/100 g, more than three times larger than that of the silica gel physical sorbent. A large amount of heat is released in the water adsorption process by CaCl_2_. The condensation heat of the water vapour is 2.443 kJ/g at room temperature; the solution heat of the CaCl_2_ hydrates such as CaCl_2_·2H_2_O, CaCl_2_·4H_2_O, and CaCl_2_·6H_2_O are 87.2, 134, and 198 J/g, respectively^18^. The CaCl_2_/silica gel composite sorbent reported by Zhu *et al.*^19^ exhibited a thermal storage capacity of up to 1 kJ/g and a storage efficiency of 0.78. Analogous to other chemical sorbents such as LiCl and LiBr, CaCl_2_ easily agglomerates; a liquid phase is formed during its adsorption process. The defects lead to a low adsorption rate of the CaCl_2_ powder. To improve the adsorption rate, CaCl_2_ has typically been added to porous media such as silica gel and activated alumina so that composite sorbents with large specific surface areas can be obtained^20^. Aristov *et al.*^21^ reported the synthesis of a CaCl_2_/silica gel composite sorbent. Its water adsorption capacity exceeded 75 g/100 g, and its desorption temperature was as low as 70 °C. By combining CaCl_2_ with the nanostructured mesoporous MCM-41, Wang *et al.*^22^ prepared a composite sorbent with 30–58 wt% CaCl_2_. The maximum water uptake of the sorbent was as high as 175 g/100 g. The water loss of the composite material could reach 90% at 80 °C. Because CaCl_2_ blocked the mass transport channels in MCM-41 for water vapour, the adsorption time for CaCl_2_ to reach sorption equilibrium exceeded 30 h. By impregnating CaCl_2_ into macroporous silica gels with an average pore size of 4.61 nm, Zhu *et al.*^19^ obtained a CaCl_2_/silica gel composite sorbent. The sorption time was less than 7 h. To increase the sorption rate, Spiridon *et al.*^23^ coated CaCl_2_ with carbon materials having huge specific surface area (680 m^2^/g) and porosity (0.27 cm^3^/g). The initial adsorption rate of the composite sorbent was 0.0275 kg/m^3^/s, and the time was approximately 2 h to reach adsorption equilibrium.

The surface adsorption processes of solid materials have been extensively studied, producing the classic monolayer adsorption model (the Langmuir model for the adsorption isotherm)^24^,^25^,^26^ and the multilayer adsorption models (the Polanyi adsorption potential theory and the Brunauer–Emmett–Teller (BET) theory)^27^,^28^. Six types of adsorption isotherms have been proposed and widely used in various research areas. Classical adsorption models are based on the adsorption processes that occur on the surface of solid materials. However, chemical sorbents such as CaCl_2_ and LiCl can rapidly adsorb water vapour, which leads to the formation of an aqueous film on the powder surface. As a result, their sorption process originates from a saturated solution. In addition, the vapour sorption process involves the diffusion of water vapour in air, the dissolution of CaCl_2_ powder, and the diffusion of water molecules, Cl^−^, and Ca^2+^ in the liquid phase. Because of these factors, classical isotherm adsorption models are not applicable to vapour sorption by chemical sorbents.

In this paper, a sorption model of solid-liquid coexistence is established based on the definition of steady-state sorption. Both theoretical analysis and experimental results indicate that the apparent sorption rate (μ) is not dependent on sorption time, suggesting that it can be modelled using a steady-state sorption model. This result implies that the ion concentration at the surface layer remains constant. It is easily understanded that the evaporation of liquid water can be viewed as a desorption process in which the ion concentration in solution is zero at all times. The evaporation equation of distilled water is similar to that of the saturated CaCl_2_ solution for the sorption process. Based on the reported evaporation equation for pure water, the steady-state sorption equation, 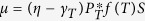, is herein proposed and validated. Based on the steady-state sorption equation, two methods have been proposed to increase the sorption rate, including increasing the specific surface area of chemical sorbent and adjusting the critical relative humidity (γ) of solution. Two types of composite sorbents have been prepared by using these methods: (1) it has been combined with carbon nanotubes to increase the specific surface area of the CaCl_2_ powder; (2) to decease the *γ* of CaCl_2_, NaCl with no sorption capability was added to the saturated CaCl_2_ solution.

## Results and Discussions

### Steady-state sorption equation

The sorption process is classified as solution sorption for CaCl_2_ chemical sorbent. It shares a similar sorption/desorption equation with the evaporation of liquid water. Dalton proposed the evaporation equation of liquid water in 1802 as following:


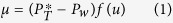


where *μ* is the sorption rate, g/(m^2^s); 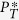, *P*_*w*_ represent the saturated vapor pressure and partial pressure of water vapour, respectively, Pa; and *f* (*u*) is the function of wind speed. Currently, the most widely used evaporation equation^29^,^30^ takes the following form:


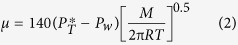


where *R* is the gas constant, J/(molK); and *T* stands for environmental temperature, K. Based on thses equation, the steady-state sorption equation for saturated solution can be deduced:


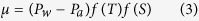


where *f* (*T*) and *f* (*S*) represent the functions of temperature and surface area of the solution, respectively, and *P*_*a*_ are the vapour pressure of the saturated solution, respectively. Because the partial pressure of water vapour (*P*_*w*_) equals the product of the relative humidity (*η*) and the saturated vapour pressure of water vapour (*P*_T_^*^), the sorption equation can be transformed to


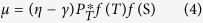


where *γ*, critical relative humidity, characterizes the critical condition of sorption/desorption of the solution. It is a function of the type and concentration of the solutes only.

**Figure 1:**
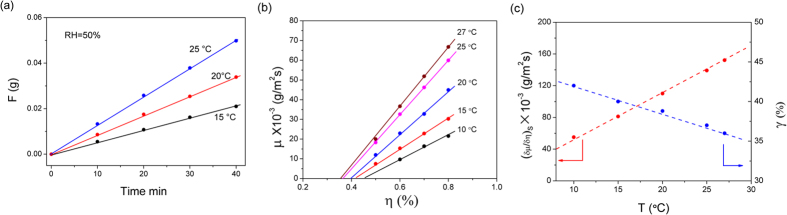
The sorption uptake of the solid-liquid phase at 15, 20 and 25 °C and relative humidity of 50%. (**a**) sorption curves; (**b**) sorption rate; and (**c**) 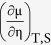
and *γ* curves.

To ensure a constant surface area of the aqueous film along with easy and accurate measurements throughout the experiment, we have employed a liquid seal for CaCl_2_ powder to construct a system of solid-liquid coexistence. The sorption of the resultant system of solid-liquid coexistence can be classified as a steady-state sorption process; the theoretical proof can be found in the Supporting Information. This conclusion is also corroborated by the isotherm sorption experiments. As shown in Fig. 1a, the sorption curves of the saturated solution were measured at 15, 20, and 25 °C under the *η* of 50%. At 20 °C, the sorption amount was 8.62, 17.5, 25.41, and 34.88 mg at a sorption time of 10, 20, 30, and 40 min, respectively. The corresponding sorption amount per unit time was 0.862, 0.875, 0.847, and 0.847 mg/min. The results indicate that the sorption rate remains constant if the testing environment remains the same. Both theoretical analysis and experimental results demonstrate that sorption in the system of solid-liquid coexistence can be classified as a steady-state sorption process. Therefore, the system of solid-liquid coexistence was modelled with the steady-state sorption equation.

### The effect of temperature and humidity on the sorption rate

To investigate the effect of temperature and humidity on the sorption rate, the sorption curves of the saturated solution were measured at different humidities and temperatures, as shown in Fig. 1b. At 20 °C, the sorption rate of the saturated solution was 10.8, 20.7, 29.5, and 40.4 mg/(m^2^s) under *η* conditions of 50%, 60%, 70%, and 80%, respectively, which indicates that the sorption rate is linearly correlated with the *η*. It can be observed from the sorption equation (Equation (4)) at constant temperature, the *γ* is a constant and the sorption rate is a linear function of the *η*. This result agrees well with the experimental data.

In particular, as shown in Fig. 1c, the *γ* of the CaCl_2_ chemical sorbent and 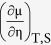
were linearly correlated with temperature and the fitting formula are:


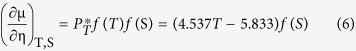



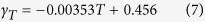


The results indicate that the sorption rate of the saturated CaCl_2_ solution is a quadratic function of temperature. The fitted equation of the steady-state sorption is as follows:


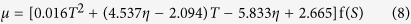


### Aqueous surface area and the rate of isothermal sorption

**Figure 2:**
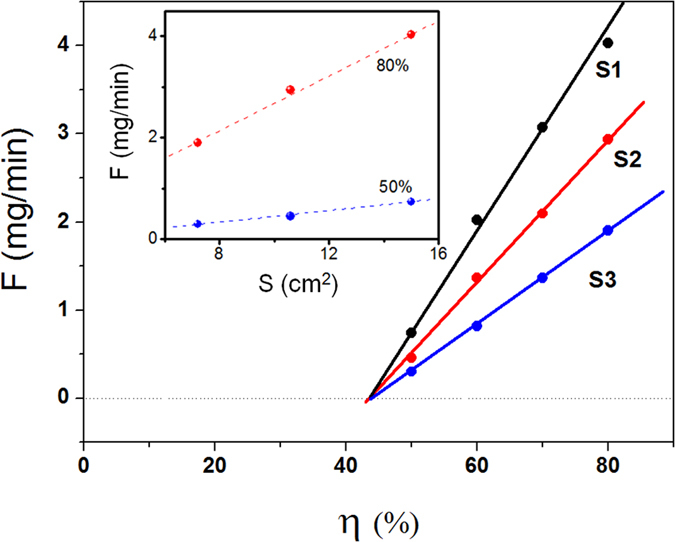
The sorption rate of CaCl_2_ saturated solutions with the liquid surface area of 15, 10.6 and 7.2 cm^2^ at 20 °C.

To investigate the relationship between the aqueous surface area and the rate of isothermal sorption, the masking method was used to adjust the aqueous surface area. As shown in Fig. 2, the sorption rate curves of three saturated CaCl_2_ solutions with different surface areas were measured. Here, S_1_ is the cross-sectional area of the beaker (15 cm^2^), and S_2_ and S_3_ are the difference between the cross-sectional areas of the beaker and the mask (10.6 and 7.2 cm^2^, respectively). It can be observed from the inset graph that under the same *η* value, the sorption rate is linearly correlated with the specific surface area. According to this relationship, the area of the mask is calculated to be 10.3 and 7.4 cm^2^, respectively, representing a decrease of only 3.0% and 2.8% compared to the measured values. This finding further confirms that the sorption rate is directly proportional to the aqueous surface area.

Based on the above results, the steady-state sorption equation is as follows:


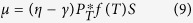


In particular, the sorption rate of the saturated CaCl_2_ solution is a quadratic function of temperature. The fitted equation of the steady-state sorption is as follows:


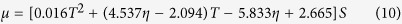


The application conditions of the equation are as follows: temperature (°C) *T* ϵ (10, 27) and *η* (%) ϵ (50, 80).

### Application of the steady-state sorption equation

It can be observed from the steady-state sorption equation (9, 10) that when both the temperature and humidity are kept constant, the sorption rate of sorbent solution can be significantly improved by increasing the surface area and decreasing the *γ* of the solution. Chemical sorbents (CaCl_2_, LiCl and LiBr) can rapidly adsorb water vapour, which leads to the formation of an aqueous sorption film on the surface of their powder. As a result, increasing the surface area of the powder will increase that of the aqueous solution. In addition, according to hydration theory^31^,^32^, hydrated salts such as NaCl and KaNO_3_ can form hydrated ion clusters in solution and can thus significantly decrease the saturated vapour pressure (*P*_*a*_) and *γ* (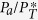). The application of the steady-state sorption equation was investigated based on these two aspects.

### Increasing the specific surface area of powder

**Figure 3:**
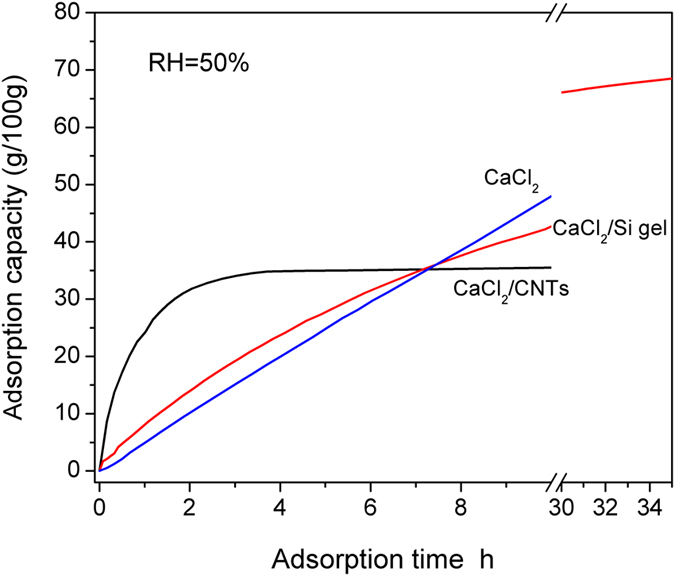
The sorption curves of the pure CaCl_2_, CaCl_2_/Si-gel and CaCl_2_/CNTs composites.

With the progression of sorption, the surface area of the chemical sorbents became progressively smaller, resulting in reduced sorption performance. To obtain CaCl_2_-based sorbents with high specific surface area and stable structure, two modification methods are reported in this paper: (1) CaCl_2_ was impregnated into silica gel to prepare an SWS-type composite sorbent (CaCl_2_/Si-gel); (2) CaCl_2_ was coated on the surface of carbon nanotubes (CNTs) to prepare a coating-modified composite sorbent (CaCl_2_/CNTs). Figure 3 shows the sorption curves of the CaCl_2_, CaCl_2_/Si-gel, and CaCl_2_/CNT composite materials. The sorption amount was 10.0, 20.0, and 29.5 g/100 g, respectively, at an sorption time of 2, 4, and 6 h. For the CaCl_2_/Si-gel composite sorbent and the same sorption time, the corresponding sorption amount was 13.8, 23.8, and 31.4 g/100 g, which was larger than the case of pure CaCl_2_ by 38%, 19%, and 6.4%, respectively. The results indicate that the CaCl_2_/Si-gel composite material enhanced sorption performance at the initial stage of sorption. However, the CaCl_2_ in the pores of the silica gel gradually formed a solution, blocking the mass transport channel of water vapour and resulting in a rapid decrease in the sorption rate. Because of this decrease, the time exceeded 35 h to reach sorption equilibrium. In contrast, the CaCl_2_/CNT sorbent can reach sorption equilibrium within 4 h, corresponding to an equilibrium sorption amount of 35.0 g/100 g. The sorption amount was 24.2, 31.8, and 34.0 g/100 g at an sorption time of 1, 2, and 3 h, respectively, accounting for 69.1%, 90.8%, and 97.1% of the sorption amount at equilibrium. Therefore, the two modification methods (improving the specific surface area of CaCl_2_ powder) can significantly increase the sorption rate at the early stage of sorption. Moreover, CaCl_2_/CNTs exhibited more outstanding performance.

**Figure 4:**
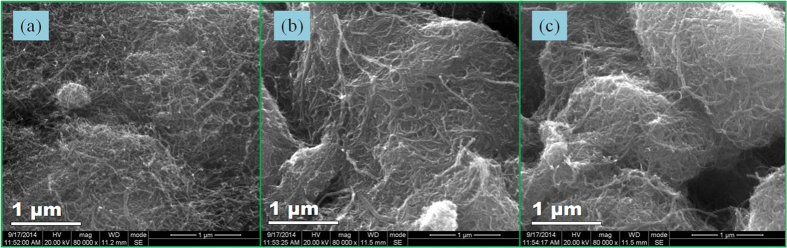
SEM of the pure CNTs (**a**) and composite. (**b**) the self-prepeared CaCl_2_/CNTs composite; and (**c**) composite material after 20^th^ cycle of sorption/desorption process.

To analyse the microstructure and stability of the coating-modified CaCl_2_/CNT sorbent, the CNTs and composite were characterised by SEM, as shown in Fig. 4. The CNTs exhibited a network structure and were uniform with a diameter of 10–20 nm. For the CaCl_2_/CNT composite sorbent, part of the CaCl_2_ was adsorbed on the surface of the CNTs, and part was deposited in the mesh resulting from the CNT network. Therefore, the surface area of the composite sorbent (48 m^2^/g) was markedly lower than that of pure CNTs (142 m^2^/g). When the CaCl_2_/CNT composite sorbent was recycled 20 times with an *η* of 50%, its microstructure did not experience any detectable change. The results indicate that the CaCl_2_/CNT composite sorbent had a very stable microstructure.

### Adjusting the critical relative humidity solution

**Figure 5:**
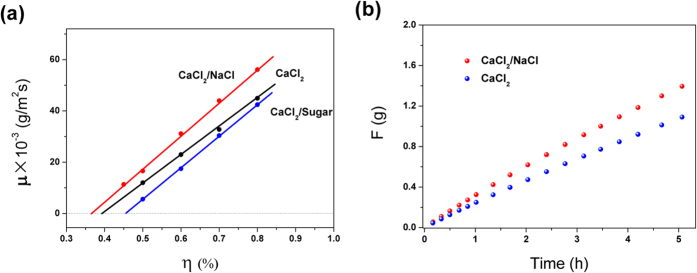
(**a**) The sorption rate of CaCl_2_, NaCl and sugar saturated solutions. (**b**) The sorption curves of CaCl_2_ and CaCl_2_/NaCl at 20 °C and relative humidity of 80%.

According to the steady-state sorption equation, the sorption performance can be significantly improved by decreasing the *γ* of the solution. The *γ* can be adjusted by the type and concentration of solutes. Based on this approach, the strong electrolyte NaCl (NaCl:CaCl_2_ solution = 1:10) and the non-electrolyte sucrose (sucrose:CaCl_2_ solution = 1:10) were used to adjust the sorption performance of the saturated CaCl_2_ solution. The testing results are shown in Fig. 5. It can be observed from Fig. 5a that when the *η* was 50%, the respective sorption rates of CaCl_2_/NaCl, CaCl_2_/sugar, and saturated solution of CaCl_2_ were 16.6, 12.0, and 5.6 g/(m^2^s), respectively. When the *η* was increased to 80%, their sorption rates increased to 56.1, 44.9, and 42.4 g/(m^2^s), respectively. Thus, the NaCl strong electrolyte improved the sorption performance, whereas the sucrose non-electrolyte decreased the sorption performance. According to hydration theory, there exist large amounts of hydrated ion clusters, Na^+^(H_2_O)_1–6_, in NaCl solution^33^,^34^. The Na^+^(H_2_O)_1–6_ clusters can decrease the molecular kinetic energy of water molecules, thus leading to the decrease of the ability of water molecules to escape from the aqueous surface. Therefore, the strong electrolyte, NaCl, can decrease the *γ* of the CaCl_2_ solution and improve its sorption rate. To further confirm these results, 0.29 and 0.28 g of CaCl_2_ were placed in the CaCl_2_ and CaCl_2_/NaCl solutions, respectively. Their sorption curves were measured under the conditions of a temperature of 20 °C and *η* of 80%, as shown in Fig. 5b. The sorption mass by the saturated CaCl_2_ solution was 0.249, 0.679, and 1.09 g, respectively, at an sorption time of 1, 3, and 5 h. For the modified CaCl_2_ solution, at the same sorption time, the corresponding sorption amount was 0.325, 0.81, and 1.39 g, respectively, which was larger than that of the saturated CaCl_2_ solution by 30.6%, 29.9%, and 27.9%. Therefore, the strong electrolyte, NaCl, can significantly improve the sorption rate of the saturated CaCl_2_ solution.

**Figure 6:**
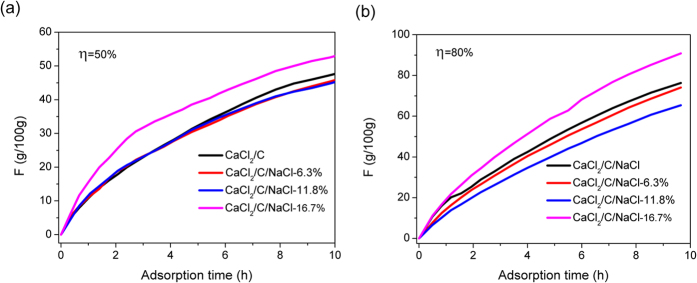
The sorption curves of CaCl_2_/C and CaCl_2_/C/NaCl*X (X* = 6.3%, 11.8% and 16.7%) at 25 °C and relative humidity of 50% (**a**) and 80% (**b**).

These experiments demonstrate that the sorption performance of saturated solution can be significantly improved by decreasing the *γ* of CaCl_2_ solution. To confirm that this method is effective for powder material, the CaCl_2_, activated carbon, and NaCl with certain ratios were homogeneously mixed to prepare a composite sorbent. The sorption curve of the resultant composite sorbent is shown in Fig. 6. The sorption performance was obviously improved for the composite with an addition of 16.7 wt% NaCl. At an *η* of 50%, the sorption amount by CaCl_2_/C (CaCl_2_:activated carbon = 1:1) samples was 23.1, 36.4, and 45.8 g/100 g at an sorption time of 3, 6, and 9 h, respectively. The sorption amount of the CaCl_2_/C/NaCl-16.7% sample was 51.0 g/100 g at 9 h, an 11.5% increase compared with that of the CaCl_2_/C sample. When the *η* was increased to 80%, the sorption amount of the CaCl_2_/C, CaCl_2_/C/NaCl-6.3%, CaCl_2_/C/NaCl-11.8%, and CaCl_2_/C/NaCl-16.7% samples at 9 h was 73.5, 71.0, 62.8, and 87.8 g/100 g, respectively. The results indicate that an appropriate amount of NaCl powder can significantly improve the sorption rate of composite materials.

### Extension of application objects

**Figure 7:**
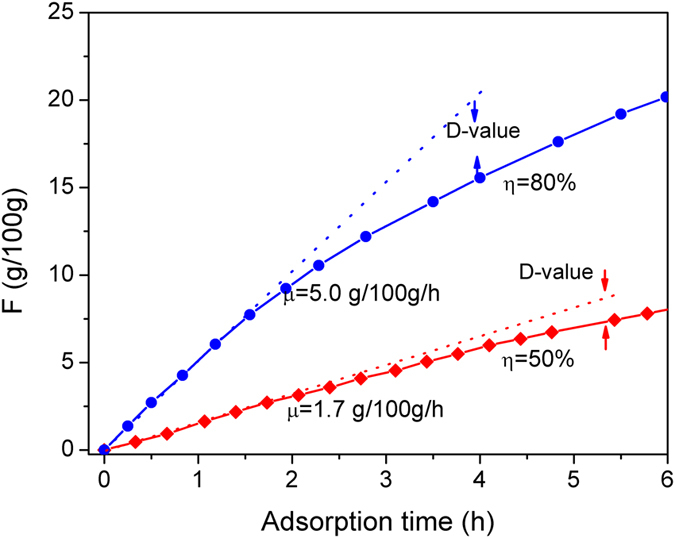
The sorption curve of copper sulphate powder (solid line), and the tangent of the sorption curve at the origin (dashed line).

The aforementioned experimental results indicate that the sorption performance can be significantly improved by increasing the specific surface area or decreasing the *γ*. To study whether the steady-state sorption equation can be applied to powder sorbents, the sorption curve of copper sulphate powder was measured, as shown in Fig. 7. The dashed line is the tangent of the sorption curve at the origin; the value of its slope, 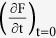, is the initial sorption rate of copper sulphate crystal. It can be observed from the figure that when *η* is equal to 50%, the testing results overlapped with the corresponding dashed line in the first 1.5 h. The results indicate that the measured sorption rate (1.7 g/100 g/h) remained constant in the first 1.5 h of the initial sorption process. Similarly, when *η* = 80%, the sorption rate of the copper sulphate crystals remained constant in the first 1.5 h of the initial sorption. These results suggest that the initial sorption process of copper sulphate powder can also be modelled as a steady-state sorption process. In addition, the measured sorption amount was markedly lower than the values corresponding to the dashed line after 1.5 h. This phenomenon occurs because the thickness increases for the water-containing crystals covering the surface of anhydrous copper sulphate, resulting in an increase of resistance to the mass transport of water molecules. Therefore, the corrected results for the steady-state sorption equation can be applied to the early-stage sorption process for the powder chemical sorbents. For the intermediate and final sorption stages, the effect of bulk mass transport on the sorption rate has to be taken into account.

## Conclusions

If the sorption rate also remains constant, the sorption process can be considered as steady-state sorption. A simple system of solid-liquid coexistence was established based on the steady-state sorption hypothesis. Through theoretical analysis and experimental results, it has been demonstrated that the sorption process of this model behaves as a steady-state sorption process. By measuring the relationship between sorption rate and testing temperature, humidity, and aqueous surface area, the steady-state sorption equation was obtained. Lastly, based on the steady-state sorption equation, two methods were proposed to increase the sorption rate, including increasing the specific surface area and adjusting the *γ* of materials.

The CaCl_2_/Si-gel and CaCl_2_/CNT composite materials dispaly higher specific surface area in comparion with that of the pure CaCl_2_ powder. For the CaCl_2_/Si-gel material, the CaCl_2_ solution formed in the sorption process blocks the pores of silica gel support, thus inducing resistance to the mass transport of water vapour. The sorption amount at 6 h was improved by only 6.4%. For the CaCl_2_/CNT composite, CaCl_2_ powder could coat the surface of the CNTs. The composite material reached sorption equilibrium within only 4 h, and the sorption capacity was improved by 75% compared with pure CaCl_2_ powder within the same time. As a result, compared with pure CaCl_2_ powder, their sorption rates were dramatically improved.

The simple method, adding NaCl with no sorption capability into the saturated CaCl_2_ solution, could effectively decrease the *γ* of solution. The sorption rate of the mixture was improved by 30% under the same testing conditions. The method was verified to be applicable to powder materials.

## Methods

### Preparation of composites

CaCl_2_ powder can rapidly adsorb water vapour in air, which leads to the formation of an aqueous film on the surface of the powder. With the progression of sorption, CaCl_2_ powder is submerged by the solution formed on its surfaces, resulting in the change of the surface area of the aqueous film. To ensure a constant surface area of the aqueous film along with easy and accurate measurements throughout the experiment, we have employed a liquid seal for CaCl_2_ powder to construct a system of solid-liquid coexistence. Briefly, an appropriate amount of CaCl_2_ powder was submerged in a saturated solution of CaCl_2_; the air contained in the powder was removed by stirring the solution. A system of solid-liquid coexistence was obtained after sitting for 12 h in order to test its sorption performances. Similarly, the systems of solid-liquid coexistence of CaCl_2_/NaCl (NaCl:CaCl_2_ solution = 1:10) and CaCl_2_/sugar (sucrose:CaCl_2_ solution = 1:10) were also prepared.

Two methods have been used to prepare the CaCl_2_ composite materials with large specific surface area. Porous silica gels were immersed in CaCl_2_ solution for 24 h, and the SWS-type CaCl_2_/Si-gel composite sorbent was obtained after being dried. In addition, CaCl_2_ and CNTs were mixed with mass ratio of 1:2.5 for the preparation of CaCl_2_/CNT composite sorbent.

Lastly, CaCl_2_, activated carbon, and NaCl were mixed with certain ratios for the preparation of CaCl_2_/C/NaCl composite sorbent. The composites with 6.3%, 11.8%, and 16.7% (mass percentage) NaCl were labelled as CaCl_2_/C/NaCl-6.3%, CaCl_2_/C/NaCl-11.8%, and CaCl_2_/C/NaCl-16.7%, respectively.

### Material characterization

The specific surface area of the composite material was measured through nitrogen sorption at 77 K using a Micro-meritics ASAP 2040 Analyzer in the range of the relative pressures P/P_0_ = 0.06–0.99 with a step of 0.015. The morphologies of the samples were observed using a field-emission scanning electron microscope (FE-SEM, Hitachi, S3400N). The sorption equilibrium between water vapour and the composite was studied in thermostat-humidistat chamber. In the thermostat-humidistat chamber, as-synthesized sorbent was put in 50 ml beakers with the cross-section of 15 cm^2^. The testing environment was set at the relative humidity of 40 to 80% at room temperature from 15 to 25 °C. The amount of water adsorbed by composite materials was measured by an electronic scale during the process until the mass reaches steady state.

## Additional Information

**How to cite this article**: Zhang, H. *et al.* Steady-state equation of water vapor sorption for CaCl_2_-based chemical sorbents and its application. *Sci. Rep.* **6**, 34115; doi: 10.1038/srep34115 (2016).

## Supplementary Material

Supplementary Information

## References

[b1] Holbach A., Bi Y., Yuan Y., Wang L. & Zheng B. Environmental water body characteristics in a major tributary backwater of the unique and strongly seasonal Three Gorges Reservoir, China. Environ. Sci.: Processes & Impacts 17, 1641–1653 (2015). 10.1039/c5em00201j 26201244

[b2] Jiang S. & Yuan Z. Phosphorus Flow Patterns in the Chaohu Watershed from 1978 to 2012. Environ. Sci. & Technol. 49, 13973–13982 (2015). 10.1021/acs.est.5b03202 26556468

[b3] Li W., Gao L., Shi Y., Liu J. & Cai Y. Occurrence, distribution and risks of antibiotics in urban surface water in Beijing, China. Environ. Sci.: Processes & Impacts 17, 1611–1619 (2015). 10.1039/c5em00216h 26245300

[b4] Mattsson K., Hansson L. A. & Cedervall T. Nano-plastics in the aquatic environment. Environ. Sci.: Processes & Impacts 17, 1712–1721 (2015). 10.1039/c5em00227c 26337600

[b5] Fairbanks R. G. A 17, 000-year glacio-eustatic sea level record: influence of glacial melting rates on the Younger Dryas event and deep-ocean circulation. Nature 342, 637–642 (1989).

[b6] Khalil A. Dehumidification of atmospheric air as a potential source of fresh water in the UAE. Desalination 93, 587–596 (1993).

[b7] Sundqvist H. A parameterization scheme for non-convective condensation including prediction of cloud water content. Q. J. Roy. Meteor. Soc. 104, 677–690 (1978).

[b8] Zhang H., Yuan Y., Yang F., Zhang N. & Cao X. Inorganic composite sorbent CaCl_2_/MWNT for water vapor sorption. RSC Advances 5, 38630–38639 (2015).

[b9] Furukawa H., Gándara F., Zhang Y. B., Jiang J. & Queen W. L. Water sorption in porous metal-organic frameworks and related materials. J. Am. Chem. Soc. 136, 4369–4381 (2014). 24588307 10.1021/ja500330a

[b10] Hasell T., Schmidtmann M., Stone C. A., Smith M. W. & Cooper A. I. Reversible water uptake by a stable imine-based porous organic cage. Chem. Commun. 48, 4689–4691 (2012). 10.1039/c2cc31212c 22476323

[b11] Wang D., Zhang J., Tian X., Liu D. & Sumathy K. Progress in silica gel–water sorption refrigeration technology. Renew. Sust. Energy Rev. 30, 85–104 (2014).

[b12] Cortés F., Chejne F., Carrasco-Marín F., Moreno-Castilla C. & Pérez-Cadenas A. Water sorption on zeolite 13X: comparison of the two methods based on mass spectrometry and thermogravimetry. Sorption 16, 141–146 (2010).

[b13] Li X. Y., Uehara M., Enomoto N. & Hojo J. Synthesis of titania-doped mesoporous silica and its gas adsorbability. J. Ceram. Soc. Jpn. 109, 818–822 (2001).

[b14] Benkoula S. Water sorption on TiO_2_ surfaces probed by soft X-ray spectroscopies: bulk materials vs. isolated nanoparticles. Sci. Rep. 5, 15088 (2015). 26462615 10.1038/srep15088 PMC4604456

[b15] Bi H. Ultrahigh humidity sensitivity of graphene oxide. Sci. Rep. 3, 2714 (2013). 24048093 10.1038/srep02714 PMC3776968

[b16] Gordeeva L. G., Grekova A. D., Krieger T. A. & Aristov Y. I. Sorption properties of composite materials (LiCl + LiBr)/silica. Micropor. Mesopor. Mat. 126, 262–267 (2009).

[b17] Kiplagat J., Wang R., Oliveira R. & Li T. Lithium chloride–expanded graphite composite sorbent for solar powered ice maker. Sol. Energy 84, 1587–1594 (2010).

[b18] Yuan Y., Zhang H., Yang F., Zhang N. & Cao X. Inorganic composite sorbents for water vapor sorption: A research progress. Renew. Sust. Energy Rev. 54, 761–776 (2016).

[b19] Zhu D., Wu H. & Wang S. Experimental study on composite silica gel supported CaCl_2_ sorbent for low grade heat storage. Int. J. Therm. Sci. 45, 804–813 (2006).

[b20] Wang J. Y., Wang R. Z. & Wang L. W. Water vapor sorption performance of ACF-CaCl_2_ and silica gel-CaCl_2_ composite sorbents. Appl. Therm. Eng. 100, 893–901 (2016).

[b21] Aristov Y. I., Restuccia G., Cacciola G. & Parmon V. A family of new working materials for solid sorption air conditioning systems. Appl. Therm. Eng. 22, 191–204 (2002).

[b22] Ji J., Wang R. & Li L. New composite sorbent for solar-driven fresh water production from the atmosphere. Desalination 212, 176–182 (2007).

[b23] Spiridon M., Hauta O. R., Secula M. S. & Petrescu S. Preparation and Characterization of Some Porous Composite Materials for Water Vapor Sorption. Revista de Chimie 63, 711–714 (2012).

[b24] Hoory S. & Prausnitz J. Monolayer sorption of gas mixtures on homogeneous and heterogeneous solids. Chem. Eng. Sci. 22, 1025–1033 (1967).

[b25] MacDonald J. R. & Barlow C. Jr Work function change on monolayer sorption. J. Chem. Phys. 39, 412–422 (1963).

[b26] Olsen S. R. & Watanabe F. S. A method to determine a phosphorus sorption maximum of soils as measured by the Langmuir isotherm. Soil Sci. Soc. Am. J. 21, 144–149 (1957).

[b27] Dubinin M. The potential theory of sorption of gases and vapors for sorbents with energetically nonuniform surfaces. Chem. Rev. 60, 235–241 (1960).

[b28] Walker W. C. & Zettlemoyer A. C. A dual-surface BET sorption theory. J. Phys. Chem. 52, 47–58 (1948). 10.1021/j150457a006 18918858

[b29] Cai L. Influence of forced air volume on water evaporation during sewage sludge bio-drying. Water Res. 47, 4767–4773 (2013). 23648285 10.1016/j.watres.2013.03.048

[b30] Pashley E. L., Zhang Y., Lockwood P. E., Rueggeberg F. A. & Pashley D. H. Effects of HEMA on water evaporation from water-HEMA mixtures. Dent. Mater. 14, 6–10 (1998). 9972145 10.1016/s0109-5641(98)00003-7

[b31] Guggenheim E. A. L. The specific thermodynamic properties of aqueous solutions of strong electrolytes. The London, Edinburgh, and Dublin Philosophical Magazine and Journal of Science 19, 588–643 (1935).

[b32] Kumar A. & Patwardhan V. S. Prediction of vapour pressure of aqueous solutions of single and mixed electrolytes. Can. J. Chem. Eng. 64, 831–838 (1986).

[b33] Uddin N. & Choi C. H. Comparative Atomic Charges on Na^+^(H_2_O)_n_ (n = 1–6) Clusters. B. Kor. Chem. Soc. 36, 827–831 (2015).

[b34] Merrill G. N., Webb S. P. & Bivin D. B. Formation of Alkali Metal/Alkaline Earth Cation Water Clusters M(H_2_O)_1−6_, M = Li^+^, Na^+^, K^+^, Mg^2+^, and Ca^2+^: An Effective Fragment Potential (EFP) Case Study. J. Phys. Chem. A 107, 386–396 (2003).

