# Supplementary material for: Steady-state equation of water vapor sorption for CaCl2-based chemical sorbents and its application
Source: Sci Rep. 2016 Sep 29;6:34115. doi: 10.1038/srep34115 (PMC5040957; doi:10.1038/srep34115)
Supplement: Supplementary Information [file srep34115-s1.pdf]

Steady-state sorption equation of $CaCl_2$-based chemical adsorbents and its application

Haiquan Zhang, Yanping Yuan*, Qingrong Sun, Xiaoling Cao and Liangliang Sun

School of Mechanical Engineering, Southwest Jiaotong University, 610031 Chengdu, China

Supporting information

Theoretical proof

If the sorption rate is constant for solution sorbent at specific temperature and relative humidity, the sorption/desorption process becomes steady-state. The solid-liquid phase coexisting system of the $CaCl_2$ chemical adsorbent can reach steady-state sorption process, theoretical proof as following:

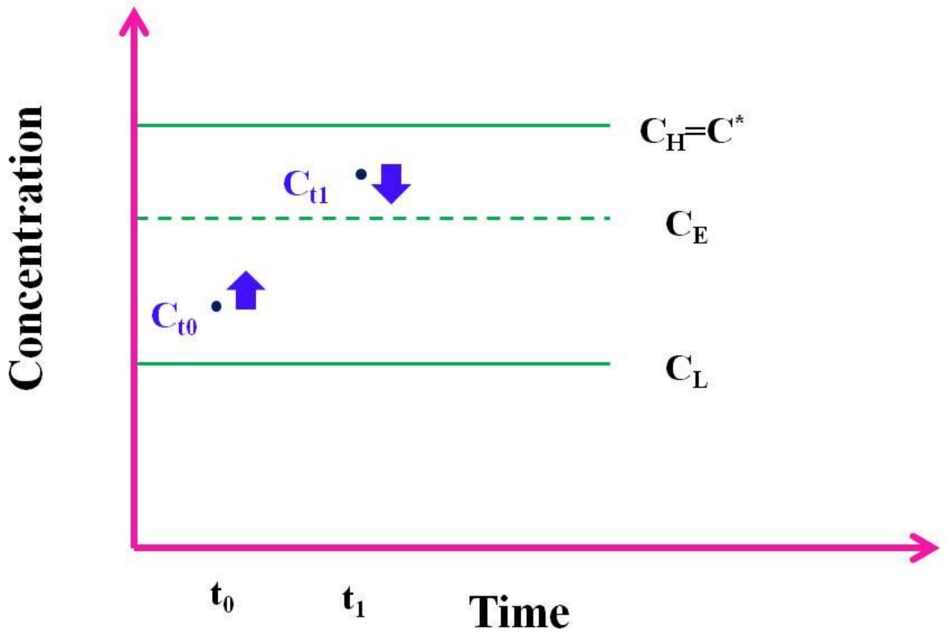

Scheme 1 Schematic of the concentration of the surface solution for the solid-liquid phase coexisting system.

When both the environmental temperature and relative humidity keep constants, the maximum concentration (C_H) of the $CaCl_2$ solution is equal to the solubility (C^*). If the environmental water

* Corresponding author. Tel.: +86 28 87634937; fax: +86 28 87634937.

E-mail address: ypyuan@home. swjtu.edu.cn.(Y. Yuan)

vapour pressure (Pa_w) is more than the solution saturated vapour pressure (Pa_t), surface solution of $CaCl_2$ can absorb water vapour. When the sorption rate is close to zero, the $CaCl_2$ solution presents the minimum value, named as C_L. For the solid-liquid phase coexisting system, the concentration (C_t) of surface solution is controlled by the water vapor sorption rate (μ_{At}) and diffusion rate (μ_{Dt}) of the Ca^{2+} and Cl^-. As we all kown, value of the C_t varies between C_H and C_L, and is a continuous function of time (t).

According to Raoult's law, the solution saturated vapour pressure Pa_t reduces as solution concentration C_t increases. It can be obviously concluded that the sorption dynamic (Pa_w -Pa_t) is an increasing function of the C_t. This means that μ_{At} is a monotonically increasing function of C_t. According to Fick's first law, diffusion rate μ_{Dt} is proportional to the concentration gradient. This means that μ_{Dt} is a monotonically decreasing function of C_t. In short, if the surface concentration C_t is increased, the μ_{At} presents a increase trend, but D-value of the μ_{Dt} will reduce. If the μ_{At} is increased and the μ_{Dt} is reduced at liquid interface, the surface concentration will display a increase trend. (***)

A hypothesis has been proposed, namely concentration of the surface solution can keep a constant (C_E) for the solid-liquid phase coexisting system. Because the μ_{At} and μ_{Dt} are monotonic function of the concentration C_t, values of the μ_{At} and μ_{Dt} are equal to constants at specific C_t, remarked as μ_{AE} and μ_{DE} respectively.

At the time of t_0, it is hypothesised that the concentration (C_{t0}) of surface solution is less than C_E. According to the derive conclusions (***), the μ_{At0} is less than μ_{AE}, but the value of the μ_{Dt0} is more than the μ_{DE}. It can be obviously concluded that the C_{t0} presents a increasing trend. On the other hand, a hypothesis has been proposed that the concentration (C_{t1}) of surface solution is over C_E at the time of t_1. The results are obtained, as following: $\mu_{At1} > \mu_{AE}$ and $\mu_{Dt1} < \mu_{DE}$. It means that the C_{t1} presents a decreasing trend during the $C_{t1} > C_E$. In summary, the value of the C_t will be closed to the C_E under the control of kinetic action, if the concentration (C_t) of surface solution is not equal to the C_E. Therefore, the sorption process becomes steady-state at specific temperature and relative humidity for a long sorption time.

When the concentration of the surface solution is a constant of C_E, the total sorption is equal to $\triangle t{*}\mu_{AE}$ during the sorption time of $\triangle t$. The $CaCl_2$ mass of diffusion at the interface is $\triangle t{*}\mu_{DE}$. Therefore, a equation is shown as below: $C_E = \frac{\Delta t * \mu_{DE}}{\Delta t * \mu_{AE}} = \frac{\mu_{DE}}{\mu_{AE}}$

Where C_E represents a mass concentration, kg/kg. The diffusion rate of calcium chloride is not easy to directly measure, but its value is can be easily calculated by using the formula of $\mu_{DE} = C_E * \mu_{AE}$.

Steady-state adsorption had been reported in published literatures.

[1] Krzan M, Zawala J, Malysa K. Development of steady state adsorption distribution over interface of a bubble rising in solutions of n-alkanols (C5, C8) and n-alkyltrimethylammonium bromides (C8, C12, C16)[J]. Colloids and Surfaces A: Physicochemical and Engineering Aspects, 2007, 298(1): 42-51.

[2] Graham D E, Phillips M C. Proteins at liquid interfaces: I. Kinetics of adsorption and surface denaturation[J]. Journal of Colloid and Interface Science, 1979, 70(3): 403-414.

[3] Ching C B, Ruthven D M. An experimental study of a simulated counter-current adsorption system—I. Isothermal steady state operation[J]. Chemical engineering science, 1985, 40(6): 877-885.

[4] Comstock G L. Moisture diffusion coefficients in wood as calculated from adsorption, desorption and steady state data[J]. Forest Products Journal, 1963, 13(3): 97-103.

Mask experiments

To investigate the relationship between the aqueous surface area (S) and the rate of isothermal sorption (μ), the masking method has been used to adjust the aqueous surface area. Low-density polyethylene (0.915~0.940 g/ml) is used as a mask, and it can float on the surface of the $CaCl_2$ solution (saturated solution, 20 ℃, 1.74 g/ml). Schematic illustration of this experimental setup is displayed as below:

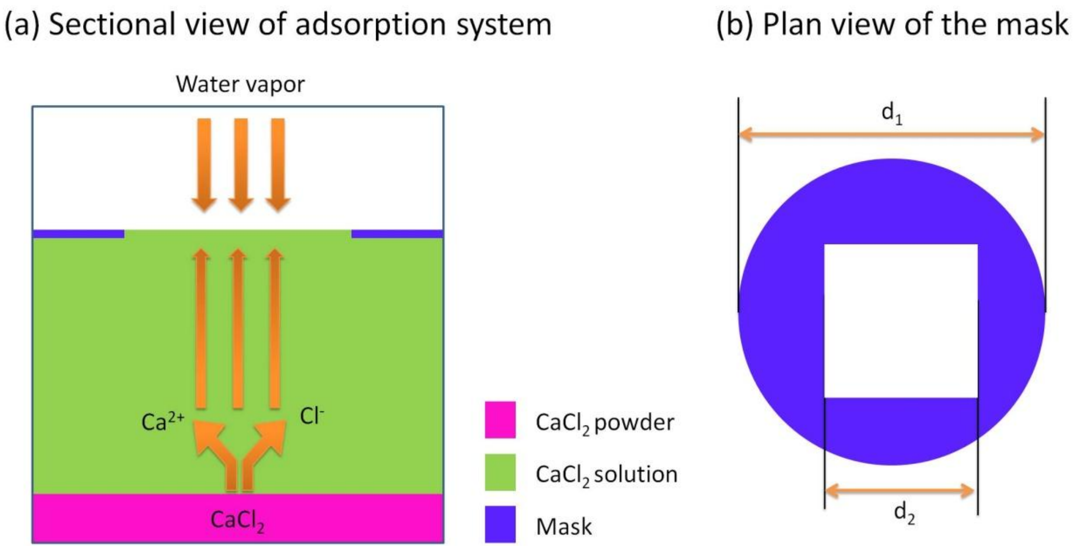

Scheme 2 Schematic illustration of the solid-liquid phase coexisting system.

The mask presents a hollow structure. Its outer diameter (d_1) is equal to the inner diameter of the beaker (4.37 cm), and its inner diameter (d_2) is 2.1 or 2.8 cm. The area of the mask is calculated to be 10.6 and 7.2 cm^2, respectively.
